# Examining the Effects of Different Types of Achievement Goal Orientation on Undergraduate Students’ Engagement in Distance Learning: The Mediating Effect of Self-Efficacy

**DOI:** 10.3390/bs15010039

**Published:** 2025-01-02

**Authors:** Tingzhi Han, Guoxing Xu, Wenli Lu

**Affiliations:** 1 School of Education, Jiangnan University, Wuxi 214122, China; 2 Institute of Higher Education, East China Normal University, Shanghai 200062, China; 3 Zhiyuan College, Shanghai Jiao Tong University, Shanghai 200240, China; luwenli@sjtu.edu.cn

**Keywords:** achievement goal orientation, online learning engagement, self-efficacy, mediating effects

## Abstract

This study investigates the impact and mechanisms of achievement goal orientation on online learning engagement among undergraduates, using a sample of 461 students enrolled in online courses from four universities of varying levels in Shanghai, China. Self-efficacy is introduced as a mediating variable, and structural equation modeling is employed to assess the effects. The findings reveal that different types of achievement goals have varying natures and intensities in their associations with undergraduate online learning engagement. Self-efficacy partially mediates the effect of mastery-approach goals on online learning engagement, but does not mediate the effects of mastery-avoidance, performance-approach, or performance-avoidance goals. The study recommends leveraging the guiding role of mastery-approach achievement goals to help students set scientific and reasonable goals, and stimulating students’ self-efficacy through the dual influences of external support and internal motivation. With the support of these strategies, significant improvements in undergraduate online learning engagement can be achieved.

## 1. Introduction

Distance education first emerged in the United States in the 1800s, when educators and students at the University of Chicago sought to connect through correspondence programs despite being in different locations (Mclsaac & Gunawardena, 1996, pp. 403–437). Online programs could be a double-edged sword, influencing students’ engagement in their learning experiences in both positive and negative ways. Existing research has shown that the primary advantage of distance education lies in its ability to overcome the spatial and temporal limitations of traditional learning methods, enabling students to access materials, attend classes, and engage in educational activities from virtually anywhere, at any time (Chow & Shi, 2014; Er Turkuresin, 2020; Turan & Gürol, 2020; A. Sun & Chen, 2016). This flexibility is especially beneficial for accommodating diverse schedules and geographical constraints, thereby broadening access to education (Turan et al., 2022). However, existing studies also highlight the challenges inherent in online programs due to the separation of time and space, which can hinder immediacy and interaction (Tang et al., 2020). The rapid advancement of internet technology has significantly contributed to the growth of synchronous online instruction, providing learners with access to platforms that facilitate real-time interactions between teachers and students. Yet, it is essential to recognize that the quality of communication in such environments can be influenced by factors such as awareness, cognitive social presence, and affective social presence. These elements often result in face-to-face classroom discussions being more effective than their online counterparts in fostering meaningful engagement and promoting learning outcomes in online courses (Chang & Hsu, 2016). The needs of the learner must be adequately addressed; otherwise, online learning will fail to produce successful results (Croft et al., 2010). Unlike traditional face-to-face classrooms, distance education often struggles to promote the depth and breadth of spontaneous interactive dialogue between students and teachers (Wut & Xu, 2021). These limitations can affect the depth of engagement and the overall effectiveness of the learning experience, ultimately constraining the scalability and impact of distance education.

Distance learning has emerged as a significant development in China, with online courses gaining substantial popularity at universities, particularly following the COVID-19 pandemic (Yao et al., 2020; Zhou et al., 2020). As a result, Chinese researchers have increasingly focused on evaluating the effectiveness of online learning and identifying areas for improvement (Zhu & Liu, 2020; Yao et al., 2020; Zhou et al., 2020). Current studies predominantly examine external factors that influence course quality, such as the development of online course platforms (T. Chen et al., 2020), improvements in course design (Jiang et al., 2020), and the enhancement of instructors’ online teaching skills (Jiang et al., 2020). While these external factors are critical for the success of online education, some researchers emphasize the need to focus more on the internal motivational factors that influence students’ learning. Broadbent and Poon (2015), in their systematic review, suggested that future research could deepen our understanding of the role of learner self-regulation in academic success within online environments, particularly through examining how motivation interacts with self-regulated learning (SRL) strategies. Motivation is a key factor for effective learning, important for both traditional and distance learners (Wighting et al., 2008). While traditional students are often more extrinsically motivated, distance learners tend to exhibit greater intrinsic motivation (Croft et al., 2010). Given this context, understanding how internal factors—such as achievement goal orientation and self-efficacy—affect students’ engagement in online learning environments is critical. These factors, which shape students’ perceptions of their academic abilities and their goals in learning, play a crucial role in motivating students to participate actively in online education.

This study aims to address the gap by focusing on the internal processes, emotions, and attitudes that influence undergraduate students’ engagement in online learning. Previous research has established that achievement goal orientation—encompassing behavioral, emotional, and cognitive dimensions—plays a critical role in shaping students’ motivation and engagement (Elliott & Dweck, 1988; Hsieh et al., 2007; Elliot & Hulleman, 2017, pp. 43–60; Miller et al., 2021). It influences how students approach learning tasks, set goals, and respond to challenges, ultimately affecting their overall engagement and performance (Karlen et al., 2019; Marjanović et al., 2019; Post & van der Molen, 2021). However, there is a relative paucity of studies examining the impact of these different types of achievement goal orientation on the engagement of Chinese students in online learning environments. Furthermore, there is still a scarcity of research on how learners’ autonomous learning influences the relationship between achievement goal orientation and undergraduate online learning engagement (Broadbent & Poon, 2015).

To address these gaps, this study introduces self-efficacy as a mediating variable between achievement goal orientation and online learning engagement among Chinese undergraduates. By examining how self-efficacy mediates the relationship between achievement goal orientation and online learning engagement, this study aims to provide a comprehensive understanding of the mechanisms through which achievement goals impact student engagement.

## 2. Literature Review and Research Hypothesis

### 2.1. Achievement Goal Orientation

Achievement goal orientation, as a cognitive framework, encapsulates individuals’ beliefs and intentions when engaging in academic or achievement-related activities. Since its inception, scholars have extensively explored the structure and components of achievement goal theory, evolving from a unidimensional to a more nuanced, multifactorial understanding. Early work in this area offered various classifications of achievement goal orientations. For instance, Nicholls (1984) differentiated between task involvement and ego involvement, while Butler and Winne (1995) proposed a division into mastery goals and performance goals. Ames (1992) expanded on this by suggesting that mastery and performance goals collectively comprise the broader construct of achievement goal orientations.

Achievement goals have traditionally been conceptualized in terms of two primary dimensions: the definition of competence and its associated valence (Elliot, 2006; Elliot & Murayama, 2008). As research in this domain has advanced, Elliot et al. (2001) further classified achievement goal orientation into four distinct types: (a) Mastery-approach goals, which emphasize personal competence and carry a positive valence; (b) Performance-approach goals, which focus on normative competence and also carry a positive valence; (c) Performance-avoidance goals, which center on normative competence but are associated with a negative valence; (d) Mastery-avoidance goals, which define competence in absolute terms and are similarly linked to a negative valence. Although an increasing number of alternative classification frameworks have been proposed to deepen our understanding of achievement goal orientation, the 2 × 2 model remains central to many studies (Miller et al., 2021). In addition, inevitably, each of these classification approaches has its own limitations and is often context-dependent, with varying degrees of applicability across different educational settings and student populations (Hunsu et al., 2021). For instance, Bong (2009) suggested that younger students may lack the cognitive awareness required to differentiate effectively between approach and avoidance goal types. However, the extent to which older students consistently exhibit and distinguish between these goal types across various learning contexts remains underexplored. Similarly, research by Murayama et al. (2011) indicates that the distinction between approach and avoidance goal types may be less salient among students from certain ethnic backgrounds.

### 2.2. Online Learning Engagement

Learning engagement refers to students’ willingness, active participation, academic focus, and emotional investment during the learning process (Pike et al., 2011). Scholars have proposed various classifications of learning engagement, with one of the most widely cited frameworks being that of Fredricks et al. (2004). Fredricks et al. noted that engagement is a multidimensional construct encompassing three core dimensions: behavioral, cognitive, and emotional engagement. Behavioral engagement pertains to active involvement in learning activities, such as attending classes, completing assignments, and participating in discussions. Cognitive engagement, on the other hand, includes students’ motivation, effort, and the use of strategies aimed at mastering content. Emotional engagement captures students’ affective responses, such as their interest, values, and emotional reactions to the learning process. These dimensions are intricately linked, with positive emotional engagement fostering greater levels of cognitive and behavioral engagement (Fredricks et al., 2004).

A robust amount of research has been conducted on engagement, especially the antecedents and outcomes of research on engagement in learning. Student engagement is influenced by a range of factors, including self-efficacy (Cai & Jia, 2020; Skinner et al., 2009), personal needs (Fredricks et al., 2004), and teacher support (Klem & Connell, 2004). Research consistently demonstrates that higher levels of engagement correlate with improved academic outcomes, such as enhanced problem-solving skills (Eseryel et al., 2014), increased persistence (Kuh et al., 2008), and greater satisfaction with the learning process (Wefald & Downey, 2009). In online learning contexts, engagement becomes even more critical, as it helps mitigate the challenges posed by limited face-to-face interaction and strengthens students’ connection to course content and peers, ultimately fostering a more enriching learning experience.

### 2.3. The Impact of Achievement Goal Orientation on Online Learning Engagement

Students’ online learning engagement is influenced by a variety of interacting psychological variables. Among these, achievement goal orientation (Elliot et al., 2001; Keys et al., 2012; Dinger & Dickhäuser, 2013; Cerasoli & Ford, 2014) and motivational components (Pintrich & De Groot, 1990; Stegers-Jager et al., 2012; Bai & Wang, 2023; Frumos et al., 2024) are particularly significant.

Empirical research has consistently shown that achievement goal orientations significantly influence undergraduate course learning outcomes. Different types of achievement goal orientations affect student learning outcomes in varying ways and may exert indirect effects through mediating variables. It was found that Chinese students with mastery goals, performance-approach goals, and performance-avoidance goals exhibit less academic procrastination, thereby effectively enhancing their levels of online learning engagement and improving online learning outcomes (Huang et al., 2019). Elliot et al. (2001) also discovered that performance-approach goals positively influence students’ exam scores, whereas performance-avoidance goals have a negative impact, mediated by exam anxiety. Despite variations in research contexts and study subjects in different countries, studies consistently demonstrate that positive responses (e.g., approach orientation and mastery orientation) within achievement goal orientations trigger positive feedback loops in student learning engagement, while negative responses (e.g., avoidance orientation and performance orientation) tend to hinder such engagement.

Based on these findings, this study proposes the following hypotheses:

**H1a.**  *Mastery-approach goal orientation positively predicts undergraduate students’ online learning engagement.*

**H1b.**  *Performance-approach goal orientation positively predicts undergraduate students’ online learning engagement.*

**H1c.**  *Mastery-avoidance goal orientation negatively predicts undergraduate students’ online learning engagement.*

**H1d.**  *Performance-avoidance goal orientation negatively predicts undergraduate students’ online learning engagement.*

### 2.4. The Mediational Role of Self-Efficacy

With the continuous advancement of internet technology, online learning has become an important mode of learning for learners in the digital age. Self-regulation learning, as a significant learning strategy, plays a crucial role in online education. Whether learners can succeed in an online learning environment largely depends on their ability to self-regulate their learning online. Managing one’s motivational beliefs, such as self-efficacy, is crucial for students’ learning regulation (Wolters, 1998, 2003).

The concept of self-efficacy was first introduced by Bandura, reflecting individuals’ judgments about their ability to accomplish tasks. Students’ learning cognition, emotions, and behaviors are influenced not only by external factors but also by internal drivers (Bandura, 1986, p. 391). As a crucial cognitive factor in the motivational process, self-efficacy not only affects individuals’ behavioral choices but also plays a decisive role in their environmental selections. In educational contexts, academic self-efficacy is widely recognized as a critical determinant of learners’ success, defined as individuals’ beliefs regarding their ability to achieve educational goals (Elias & MacDonald, 2007; Morelli et al., 2023). In the context of academic self-efficacy, Bandura et al. (1996) emphasized the importance of self-efficacy in self-regulated learning, which refers to the belief in one’s capacity to effectively employ self-regulation strategies during learning tasks. This construct becomes particularly salient as individuals progress into adolescence and beyond, given the increasing need for autonomy in learning (Cattelino et al., 2019).

Recent studies have increasingly focused on the dynamics of self-efficacy, particularly its antecedents and subsequent outcomes. These works examine how various internal and external influences, such as self-regulated learning (Morelli et al., 2021), attributional feedback (Coffee & Rees, 2011), and social comparison (Li, 2019), shape individuals’ self-efficacy. Some empirical studies to date have also indicated the impact of achievement goal orientation on self-efficacy (Soyer & Kirikkanat, 2019; Dull et al., 2015). Achievement goal orientation theory suggests that in goal-pursuit activities, goal expectations not only play an organizational role during the activity but also serve as evaluative criteria for the goal orientation within the activity. Social cognitive theory posits that self-efficacy is often derived from individuals’ meaningful evaluations of their performance through social comparisons with others or self-referential comparisons. This suggests that different achievement goal orientations can lead to varying levels of self-efficacy. Carmen et al. (2008) found that different types of achievement goal orientations have varying effects on students’ self-efficacy. Some studies have found that approach goals, both mastery and performance, positively predict academic self-efficacy (Zhang et al., 2022; W. Sun & Huang, 2019). However, the effects of avoidance goals on academic self-efficacy remain inconsistent (Zhang et al., 2022).

Additionally, a growing body of literature has explored the far-reaching consequences of self-efficacy, demonstrating its critical impact on academic achievement (Hwang et al., 2016; Honicke & Broadbent, 2016), persistence (Cardon & Kirk, 2015), and overall learning outcomes (P. L. Chen et al., 2023). These findings highlight the multifaceted nature of self-efficacy and its pivotal role in educational settings. Furthermore, some researchers have examined the impact of self-efficacy on learning engagement from the perspective of controllable-action expectations. In these studies, the control-value model has shown significant effects. Control–value theory, based on expectancy–value motivation theory, posits that the value and controllability of learning activities influence learning engagement, with self-efficacy being a key indicator of activity controllability. The model finds that self-efficacy positively predicts learning engagement, where high or low self-efficacy unconsciously activates positive or negative emotions through controllability assessments, thereby affecting learning engagement (Zhong et al., 2023). Chinese researchers have also found that self-efficacy is a positive predictor of online learning engagement (Cai & Jia, 2020).

The present study hypothesizes that undergraduate students’ self-efficacy may serve as an important mediating variable in the relationship between achievement goal orientations and online learning engagement. Based on these insights, the following hypotheses are proposed:

**H2a.**  *Mastery-approach goal orientation positively influences undergraduate students’ self-efficacy.*

**H2b.**  *Performance-approach goal orientation positively influences undergraduate students’ self-efficacy.*

**H2c.**  *Mastery-avoidance goal orientation positively influences undergraduate students’ self-efficacy.*

**H2d.**  *Performance-avoidance goal orientation negatively influences undergraduate students’ self-efficacy.*

**H3.**  *Self-efficacy positively predicts undergraduate students’ online learning engagement.*

**H4a.**  *Self-efficacy mediates the impact of mastery-approach goal orientation on undergraduate students’ online learning engagement.*

**H4b.**  *Self-efficacy mediates the impact of performance-approach goal orientation on undergraduate students’ online learning engagement.*

**H4c.**  *Self-efficacy mediates the impact of mastery-avoidance goal orientation on undergraduate students’ online learning engagement.*

**H4d.**  *Self-efficacy mediates the impact of performance-avoidance goal orientation on undergraduate students’ online learning engagement.*

## 3. Methodology

### 3.1. Participants

Ethical endorsement for our study was obtained. During the data collection processes, we strictly followed ethical principles. In the formal data collection, this study employed a stratified purposive sampling strategy to invite undergraduate students enrolled in online courses from four different levels of universities in Shanghai to complete an online questionnaire. A total of 476 questionnaires were collected, and after excluding invalid responses, 461 valid questionnaires were obtained, resulting in an effective response rate of 96.8%.

Regarding gender distribution, there were 313 females, accounting for 67.9%, and 148 males, accounting for 32.1%. In terms of academic disciplines, there were 262 students in humanities and social sciences, comprising 56.8%, and 199 students in STEM (science, technology, engineering, and mathematics) and medical fields, comprising 43.2%. As for grade distribution, the numbers of students from freshman to senior years and above were 131, 180, 97, 45, and 8, respectively, which corresponds to 28.4%, 39.0%, 21.0%, 9.8%, and 1.7%. Generally, students tend to take fewer elective courses as they advance in their academic years, a trend reflected in the grade distribution of the surveyed participants in this study. Additionally, the sample included undergraduate students from various types of institutions, with 60.5% attending top-tier universities and 39.5% enrolled in ordinary-level institutions. While the sample size of 476 participants may limit the generalizability of the findings, it includes a diverse range of students from different academic disciplines, university types, and grade levels. This diversity makes the sample a useful representation of undergraduate students enrolled in online courses across a variety of institutions in Shanghai.

### 3.2. Measurement Instruments

#### 3.2.1. Achievement Goal Orientation Scale

This study utilized the Achievement Goal Orientation Scale developed by Elliot et al. in 2001 to measure achievement goal orientations. The scale integrates the 2 × 2 matrix model of achievement goal orientations based on existing research (Elliot et al., 2001). Each of the scale’s four dimensions comprises three items. Respondents rated each item on a 5-point Likert scale ranging from 1 (“completely disagree or rarely agree”) to 5 (“completely agree or almost completely agree”). Higher scores indicate stronger achievement goal orientations.

#### 3.2.2. Self-Efficacy Scale

Self-efficacy reflects individuals’ judgments of their capabilities to perform specific tasks, with a particular focus on undergraduate students’ academic self-efficacy in online learning for this study. In the present study, we employed the self-efficacy subscale from the Chinese version of the Motivated Strategies for Learning Questionnaire (MSLQ-RCV), which consists of seven items. The MSLQ-RCV is a revised adaptation of the original Motivated Strategies for Learning Questionnaire developed by Pintrich and has been extensively utilized in educational research (Schunk, 2005; Moos & Ringdal, 2012; Roth et al., 2016). Specifically, the self-efficacy subscale has become one of the most frequently used instruments for assessing learners’ self-efficacy (Honicke & Broadbent, 2016). In the Chinese context, this subscale was further adapted by Lee et al. (2010) in the Learning Motivation Strategy Questionnaire, which has been widely employed in studies involving Chinese populations (Lee et al., 2010). The questionnaire comprises 7 items rated on a 5-point Likert scale, where 1 indicates “completely disagree or rarely agree” and 5 indicates “completely or almost completely agree”. Higher scores indicate higher levels of academic self-efficacy in online learning among undergraduate students.

#### 3.2.3. Online Learning Engagement Scale

The Online Learning Engagement Scale was chosen for this study because it is a well-established tool that effectively measures the three key dimensions of student engagement: behavioral, emotional, and cognitive engagement. The scale was adapted from the original version developed by Fredricks et al. (2004), which has been widely used to assess school engagement, but was modified by J. C. Y. Sun and Rueda (2012) to suit the context of Chinese students in online and distance learning environments. In this study, we adapted the engagement scale developed by Sun and Rueda and conducted a preliminary test, which revealed internal consistency coefficients of 0.720 for emotional engagement, 0.832 for behavioral engagement, and 0.416 for cognitive engagement. To enhance internal consistency, we removed specific items, resulting in a revised scale comprising 13 items: 4 for cognitive engagement, 3 for behavioral engagement, and 6 for emotional engagement. Responses were measured on a 5-point Likert scale ranging from 1 (“completely disagree or rarely agree”) to 5 (“completely agree or almost completely agree”), with higher scores indicating greater levels of engagement in each dimension.

### 3.3. Reliability and Validity Testing of Measurement Instruments

The items of the final instruments are described in Appendix A. Reliability and validity tests were conducted on the measurement instruments used in this study, as shown in Table 1.

Cronbach’s alpha coefficients were employed to assess internal consistency. The results indicated that the Achievement Goal Orientation Scale, Self-Efficacy Scale, and Online Learning Engagement Scale all had Cronbach’s alpha coefficients exceeding 0.80, indicating good reliability. Based on this, further reliability tests were conducted on the four subscales of achievement goal orientation: Mastery-Approach Goals, Mastery-Avoidance Goals, Performance-Approach Goals, and Performance-Avoidance Goals. The results revealed Cronbach’s alpha coefficients of 0.823, 0.892, 0.884, and 0.901, respectively. Reliability tests were also performed on the three subscales of Online Learning Engagement: emotional engagement, behavioral engagement, and cognitive engagement, with Cronbach’s alpha coefficients of 0.720, 0.832, and 0.964, respectively, indicating good reliability.

The Average Variance Extracted (AVE) values for each scale were 0.685, 0.727, and 0.833, respectively, all exceeding 0.5, demonstrating good convergent validity (Hair et al., 2010, pp. 599–638). Furthermore, the square root of AVE values for each scale exceeded the corresponding Pearson correlation coefficients, as shown in Table 1, indicating good discriminant validity among the three measurement tools.

In summary, the measurement instruments utilized in this study exhibited good reliability and validity.

## 4. Results

### 4.1. Common Method Bias Test

Given that the data in this study were all self-reported by undergraduates, there is a potential for common method bias. To address this issue, Harman’s single-factor test was employed to statistically assess the presence of common method bias. The results indicated that the variance explained by the first principal factor was 28.455%, which is below the 40% threshold. Therefore, the data in this study do not exhibit significant common method bias.

### 4.2. Descriptive and Correlational Analysis of Variables

Table 2 presents the means, standard deviations, and correlation coefficients for each variable in this study. The results reveal that both mastery-approach goals and performance-approach goals are significantly positively correlated with undergraduate students’ online learning engagement, while mastery-avoidance goals and performance-avoidance goals are significantly negatively correlated with online learning engagement. Furthermore, mastery-approach goals and performance-approach goals show significant positive correlations with self-efficacy, whereas mastery-avoidance goals and performance-avoidance goals negatively impact undergraduates’ self-efficacy. Additionally, self-efficacy is significantly positively correlated with online learning engagement.

### 4.3. Path Analysis and Hypothesis Testing

Q-Q plots were utilized to evaluate the normality of the data, which were determined to conform to a normal distribution. SEM in AMOS 24.0 was used to analyze the relationships among four types of achievement goals, self-efficacy, and online learning engagement, and to test the hypotheses (Byrne, 2016). Table 3 presents the results.

This study hypothesized that different achievement goal orientations (H1a, H1b, H1c, and H1d) would influence online learning engagement (OLE). The analysis results reveal that mastery-approach goals (MAP) significantly affect OLE (β = 0.166, *p* = 0.010), supporting H1a. However, performance-approach goals (PAP) (β = 0.060, *p* = 0.317), mastery-avoidance goals (MAV) (β = −0.077, *p* = 0.141), and performance-avoidance goals (PAV) (β = −0.049, *p* = 0.278) do not significantly affect OLE, thus not supporting H1b, H1c, and H1d. Furthermore, the influence of achievement goal orientations on self-efficacy (SE) was hypothesized (H2a, H2b, H2c, and H2d). The results show that mastery-approach goals (MAP) (β = 0.335, *p* < 0.001), performance-approach goals (PAP) (β = 0.415, *p* < 0.001), and mastery-avoidance goals (MAV) (β = 0.116, *p* = 0.006) significantly affect SE, supporting H2a, H2b, and H2c. However, performance-avoidance goals (PAV) do not significantly affect SE (β = −0.065, *p* = 0.188), thus not supporting H2d. Lastly, the positive effect of self-efficacy on online learning engagement (OLE) is supported (β = 0.449, *p* < 0.001), confirming H3.

### 4.4. Mediation Effect Test

To further test the significance of the mediating effect, a bias-corrected percentile bootstrap procedure (5000 resamples) was used (Preacher & Hayes, 2008). As shown in Table 4, self-efficacy played a partial mediating role in the effect of mastery-approach goals on online learning engagement, with a 95% confidence interval of [0.067, 0.195], the mediation effect proportion is 37.870%. Hypothesis H4a was supported. In contrast, self-efficacy did not demonstrate a significant mediating effect in the relationships between the other three types of achievement goal orientations (performance-approach, performance-avoidance, and mastery-avoidance goals) and online learning engagement. Hypotheses H4b, H4c, and H4d were not supported.

## 5. Discussion

### 5.1. The Impact of Achievement Goals on Engagement in Online Learning

From the perspective of the path analysis of achievement goals on undergraduate students’ online learning engagement, this study underscores that among the four types of achievement goal orientations, only mastery-approach significantly and positively predicts online learning engagement. These findings are consistent with previous research by Elliot et al. (2011), which also emphasized the positive impact of mastery-approach goals and the negative influence of avoidance-oriented goals on engagement. Based on the study of Elliott et al., this study further finds the differences in the direct-action paths of between two types of mastery-approach objectives, namely, mastery-approach and mastery-avoidance with online learning engagement.

Furthermore, the study found that self-efficacy did not always mediate the effects of all four different types of achievement goals on online learning engagement. Specifically, only mastery-approach goals positively influence online learning engagement via self-efficacy. This aligns with prior work suggesting that mastery-approach goals enhance self-efficacy, which, in turn, boosts engagement (Schunk & Greene, 2018, pp. 19–35). However, mastery-avoidance, performance-approach, and performance-avoidance goals do not significantly affect online learning engagement through this mediating pathway. This reinforces the notion that mastery-approach goals have the most substantial impact on fostering engagement, consistent with findings from previous studies (Huang et al., 2019).

This study provides a more nuanced understanding of how mastery goal orientation influences online learning engagement, extending beyond the conclusions of previous research. While much of the literature consistently demonstrates that mastery goal orientation is a positive predictor of students’ learning engagement (Elliot et al., 2001; Huang et al., 2019), the present study reveals a more complex relationship between specific types of mastery goals and online learning engagement. Specifically, it found that mastery-approach goals are positively associated with students’ engagement, while mastery-avoidance goals exert no positively associated influence. This distinction between the two types of mastery goals provides a deeper insight into how goal orientation shapes students’ behaviors in digital learning contexts.

### 5.2. The Mechanism of Self-Efficacy and Its Mediating Role

The developmental mechanism of self-efficacy provides a comprehensive theoretical framework for understanding its complete mediating role in the relationship between achievement goal orientations and engagement in online learning. Grounded in Albert Bandura’s social cognitive theory, self-efficacy is recognized as a pivotal factor influencing how individuals’ approach and persist in their learning tasks (Bandura, 1997, pp. 4–6). Schunk and Zimmerman’s model of self-regulation further elucidates how self-efficacy develops through learning processes. This model highlights that learners engage in educational activities through observation, imitation, self-control, and self-regulation. Initially, external environmental factors, such as supportive teachers and positive reinforcement, play a crucial role in building self-efficacy. These supports help learners develop confidence in their abilities. As learners progress, intrinsic factors, such as personal motivation and self-belief, become increasingly influential in sustaining and enhancing self-efficacy. Self-regulation, a key aspect of this model, refers to the learners’ ability to set goals, monitor progress, and adjust strategies based on feedback and self-assessment. Through effective self-regulation, self-efficacy can significantly influence and guide learning behaviors, enabling individuals to adapt their learning strategies to meet challenges and achieve their academic goals (Schunk & Greene, 2018, pp. 19–35).

The findings of this study underscore the significant role of self-efficacy in mediating the impact of different types of achievement goal orientations on undergraduate students’ online learning engagement. Specifically, the study reveals that self-efficacy positively mediates the relationship between mastery-approach goals and students’ online learning engagement. This finding aligns with previous research by Bandura (1997, pp. 4–6), which highlighted how self-efficacy beliefs enhance engagement, persistence, and learning strategies. When students pursue mastery-approach goals, which focus on mastering content and improving skills, their self-efficacy beliefs are strengthened, leading to increased motivation, persistence, and engagement in online learning activities. In contrast, the study indicates that self-efficacy does not mediate the relationship between the other three types of goal orientations (mastery-avoidance, performance-approach, and performance-avoidance) and students’ online learning engagement. This observation is in line with previous studies suggesting that avoidance goals tend to be less conducive to engagement because they are focused on avoiding failure rather than achieving mastery (Elliot et al., 2001; Zhang et al., 2022). For instance, mastery-avoidance goals, which focus on avoiding the negative consequences of failure, often result in lower self-efficacy due to frequent setbacks, reinforcing disengagement. Similarly, performance-approach goals, which center on outperforming others, may not sustain deep engagement, as they often focus on external validation rather than intrinsic motivation (Ames, 1992). While performance-approach goals may lead to short-term increases in engagement, they do not necessarily foster the same level of sustained motivation and effort that mastery-oriented goals do (Schunk & Greene, 2018, pp. 19–35).

Previous research has highlighted the mediating role of self-efficacy in traditional offline classroom settings, demonstrating its crucial influence on students’ academic engagement and performance (Honicke et al., 2019; Galyon et al., 2012; Gore, 2006). The mediating effect of self-efficacy is particularly pronounced in online learning environments, where students face unique challenges such as the need for higher self-regulation and increased independence. This study reaffirms the critical role of self-efficacy in mediating the impact of achievement goal orientations on students’ online learning engagement (W. Sun & Huang, 2019; Cai & Jia, 2020; Zhong et al., 2023). In the online learning context, students’ online learning engagement can be improved by generating self-efficacy under the action of mastery-approach goals.

## 6. Conclusions and Implications

### 6.1. Promoting Mastery-Approach Achievement Goals

Promoting mastery-approach achievement goals is crucial for fostering self-efficacy, learning engagement, and long-term academic success, especially in the online education environment. These goals prioritize personal growth and skill development over competitive outcomes. To encourage mastery-approach goals, educators should create a learning environment that values effort, persistence, and the process of learning, rather than focusing solely on grades or comparison with others.

One effective strategy is to incorporate formative assessments, which provide ongoing feedback on student progress without the pressure of final grades. This allows students to identify areas for improvement, build confidence, and focus on learning as a continuous journey. Shifting from summative assessments to formative feedback helps students set personal learning goals and track their progress, aligning with mastery-oriented values. Instructional strategies such as differentiated instruction are also key. Tailoring lessons to students’ individual learning styles and paces fosters a sense of ownership over their learning. When students feel that the material is accessible and matched to their abilities, they are more likely to engage deeply, strengthening their motivation to master content rather than simply achieve high grades. In addition, promoting self-regulation is essential. Encouraging students to set their own learning objectives, reflect on their progress, and assess their strengths and weaknesses builds their sense of competence and autonomy. Teachers can support this by offering resources for self-monitoring and reflection, helping students develop the skills needed for lifelong learning.

Ultimately, fostering mastery-approach achievement goals empowers students to adopt a growth mindset, where learning is valued for its own sake. This shift not only enhances academic performance but also helps students become lifelong learners, resilient in the face of challenges, and motivated by personal growth rather than external validation. By embedding these principles into classroom practice, educators can cultivate an environment that supports both academic success and holistic development.

### 6.2. Fostering Undergraduates’ Self-Efficacy

From the perspective of self-efficacy development, external environmental support is a prerequisite for learners’ self-efficacy formation, while intrinsic motivation serves as an inexhaustible driving force for sustained self-efficacy growth. Therefore, universities must focus on providing high-quality support within the online learning environment. This support extends beyond robust course platforms and technical tools. The key to ensuring effective online teaching lies in the thoughtful design of the curriculum. In traditional face-to face instruction, many educators adhere to “transmissive” and “cramming” lecture modes. The sudden emergence of online education caught many educators unprepared, resulting in insufficient preparation for online teaching, outdated online teaching models, and a lack of immediacy in online teaching.

In the online classroom, instructors need to facilitate learners’ construction of knowledge by providing scaffolded learning experiences, guiding students through specific problem-solving contexts. Under the direction and inspiration of the teacher, students should be encouraged to engage in independent exploration. Increased communication and interaction within the online classroom allow learners to absorb new perspectives, acquire fresh knowledge, and broaden their intellectual horizons. This, in turn, enhances students’ interest in learning, improves their online learning experience, and leads to satisfaction stemming from their academic success.

Intrinsic learning goals and motivation are far more conducive to the sustainable development of learners’ self-efficacy than external rewards or incentives. However, intrinsic motivation does not emerge automatically; it requires deliberate educational guidance. In the context of online education, instructors should set specific, achievable learning goals that reflect students’ individual developmental needs, taking into account their current abilities as well as their potential for growth. Such an approach motivates students to take an active role in their learning, unlocking their potential and enabling them to surpass their current academic capabilities. When students receive effective external support in the form of educational resources, they are more likely to internalize academic goals and intrinsic motivations, which ultimately fosters the development of self-efficacy. Empowered by internal motivation, learners gain the capacity for self-organization, self-management, self-assessment, and self-regulation, all of which contribute to greater engagement in online learning and improvements in learning outcomes.

### 6.3. Limitations and Future Research

While this study provides valuable insights into the engagement of undergraduate students in online learning, it is important to note some limitations. Firstly, the sample size of 476 participants may not adequately reflect the broader population of undergraduate students in Shanghai. The sample was drawn from four universities, and while efforts were made to include a range of academic disciplines and university types, the findings may not be easily generalizable to all institutions or to other regions. Additionally, the cross-sectional nature of the data limits our ability to draw conclusions about causal relationships between achievement goal orientations and student engagement. Future research with larger, more diverse samples and longitudinal designs could provide a more comprehensive understanding of these relationships.

## Figures and Tables

**Table 1:** Reliability and Validity Testing of Measurement Instruments.

Variable	Convergent Validity		Discriminant Validity		
	Cronbach’s Alpha	AVE	Achievement Goal Orientation	Self-Efficacy	Online Learning Engagement
Achievement Goal Orientation	0.872	0.685	**0.828**		
Self-Efficacy	0.923	0.727	0.489	**0.853**	
Online Learning Engagement	0.900	0.833	0.403	0.497	**0.913**

Note: In the discriminant validity table, the diagonal cells in bold represent the square root of the AVE values, and the lower triangle represents the Pearson correlation coefficients.

**Table 2:** Descriptive Statistics and Correlation Coefficients among Variables.

Variable	Mean	Standard Deviation	Correlation Coefficients					
			1	2	3	4	5	6
1. Mastery-Approach Goals	3.768	0.755	—					
2. Mastery-Avoidance Goals	3.651	0.772	0.560 ***	—				
3. Performance-Approach Goals	3.722	0.781	0.681 ***	0.457 ***	—			
4. Performance-Avoidance Goals	3.510	0.803	0.428 ***	0.504 ***	0.397 ***	—		
5. Self-Efficacy	3.480	0.744	0.521 ***	−0.348 ***	0.551 ***	−0.342 ***	—	
6. Online Learning Engagement	3.572	0.756	0.432 ***	−0.330 ***	0.415 ***	−0.298 ***	0.497 ***	—

Note: *** indicates *p* < 0.001; — indicates no correlation for that variable with itself (on the diagonal).

**Table 3:** Path coefficients analysis.

X→Y	Estimate (B)	S.E.	C.R.	*p*	β	Result
MAP→OLE	0.500	0.193	2.590	0.010	0.166	H1a supported
PAP→OLE	0.174	0.174	1.000	0.317	0.060	H1b not supported
MAV→OLE	−0.225	0.153	−1.473	0.141	−0.077	H1c not supported
PAV→OLE	−0.137	0.127	−1.084	0.278	−0.049	H1d not supported
MAP→SE	0.330	0.058	5.704	0.000	0.335	H2a supported
PAP→SE	0.395	0.051	7.746	0.000	0.415	H2b supported
MAV→SE	0.107	0.039	2.746	0.006	0.116	H2c supported
PAV→SE	−0.062	0.047	−1.317	0.188	−0.065	H2d not supported
SE→OLE	1.370	0.150	9.138	0.000	0.449	H3 supported

Note: MAP—Mastery-approach goal orientation; PAP—Performance-approach goal orientation; MAV—Mastery-avoidance goal orientation; PAV—Performance-avoidance goal orientation; SE—Self-efficacy; OLE—Online learning engagement.

**Table 4:** Mediation test.

		Effect Value	Std. Error	BC 95% CI		*p*-Value	Proportion of Mediation Effect
				Lower Bound	Upper Bound		
	Indirect effect	0.143	0.033	0.067	0.195	0.000	37.870%
MAP ⇒ SE ⇒ OLE	Direct effect	0.235	0.078	0.082	0.388	0.003	
	Total effect	0.379	0.079	0.224	0.534	0.000	
	Indirect effect	−0.158	0.040	−0.074	0.228	0.431	
PAP ⇒ SE ⇒ OLE	Direct effect	0.076	0.070	−0.062	0.214	0.279	-
	Total effect	−0.234	0.070	−0.097	0.370	0.051	
	Indirect effect	−0.020	0.020	−0.059	0.020	0.316	
MAV ⇒ SE ⇒ OLE	Direct effect	0.105	0.062	−0.017	0.226	0.093	-
	Total effect	0.085	0.065	−0.043	0.212	0.195	
	Indirect effect	0.040	0.019	0.002	0.079	0.051	
PAV ⇒ SE ⇒ OLE	Direct effect	−0.003	0.052	−0.105	0.098	0.947	-
	Total effect	0.036	0.054	−0.070	0.142	0.504	

## Data Availability

Data are contained within the article.

## Appendix A

**Achievement goal orientation:** 

1. It is important for me to do better than other students.
2. It is important for me to do well compared to others in this class.
3. My goal in this class is to get a better grade than most of the other students.
4. I worry that I may not learn all that I possibly could in this class.
5. Sometimes I’m afraid that I may not understand the content of this class as thoroughly as I’d like.
6. I am often concerned that I may not learn all that there is to learn in this class.
7. I want to learn as much as possible from this class.
8. It is important for me to understand the content of this course as thoroughly as possible.
9. I desire to completely master the material presented in this class.
10. I just want to avoid doing poorly in this class.
11. My goal in this class is to avoid performing poorly.
12. My fear of performing poorly in this class is often what motivates me.

**Self-efficacy:** 

1. Compared to other students in this class, I expected to do well.
2. I am certain that I can understand the ideas taught in my classes.
3. Compared with others in this class, I think I am a good student.
4. I am sure I can do an excellent job with the class assignments and homework.
5. I think I will receive good grades in my exams.
6. My study skills are excellent compared with others in this class.
7. I know that I will be able to learn the materials for the tests and exams.

**Online Learning Engagement** 

1. I follow the rules of the online class.
2. I complete my homework on time.
3. I have trouble using the online class.
4. I like taking the online class.
5. I feel excited by my work at the online class.
6. The online classroom is a fun place to be.
7. I am interested in the work at the online class.
8. I feel happy when taking online class.
9. I feel bored by the online class.
10. I study at home, even when I do not have a test.
11. I try to look for some course-related information on other resources such as television, journal papers, magazines, etc.
12. When I read the course materials, I ask myself questions to make sure I understand what it is about.
13. If I do not know about a concept when I am learning in the online class, I do something to figure it out.
